# Effect of patterned polyacrylamide hydrogel on morphology and orientation of cultured NRVMs

**DOI:** 10.1038/s41598-018-30360-6

**Published:** 2018-08-10

**Authors:** I. Sanzari, E. J. Humphrey, F. Dinelli, C. M. Terracciano, T. Prodromakis

**Affiliations:** 1 University of Southampton, Nanoelectronics & Nanotechnology Research Group, Electronics and Computer Science, Southampton, SO17 1BJ UK; 2 National Heart and Lung Institute, Imperial College London, London, UK; 3 Consiglio Nazionale delle Ricerche (CNR), INO UOS ‘A. Gozzini’ Area della Ricerca di Pisa - S. Cataldo, via Moruzzi 1, I-56124 Pisa, Italy

## Abstract

We recently demonstrated that patterned Parylene C films could be effectively used as a mask for directly copolymerizing proteins on polyacrylamide hydrogel (PAm). In this work, we have proved the applicability of this technique for studying the effect such platforms render on neonatal rat ventricular myocytes (NRVMs). Firstly, we have characterised topographically and mechanically the scaffolds in liquid at the nano-scale level. We thus establish that such platforms have physical properties that closely mimics the *in vivo* extracellular environment of cells. We have then studied the cell morphology and physiology by comparing cultures on flat uniformly-covered and collagen-patterned scaffolds. We show that micro-patterns promote the elongation of cells along the principal axis of the ridges coated with collagen. In several cases, cells also tend to create bridges across the grooves. We have finally studied cell contraction, monitoring Ca^2+^ cycling at a certain stimulation. Cells seeded on patterned scaffolds present significant responses in comparison to the isotropic ones.

## Introduction

The native cardiac tissue is characterised by a high anisotropic architecture that is supported by the parallel alignment of cells. The extracellular matrix (ECM) of the myocardium provides structural and biochemical cues helping cell organisation in a contractile tissue. The anisotropic architecture facilitates the preferential propagation of the electrical signal in one direction^1^ and increases the conduction velocity along the tissue^2^. A wide range of soft materials, such as elastomers or hydrogels, was exploited to date for synthesising the artificial ECM, with hydrogels gaining significant interest in cardiac tissue engineering^3^. These materials have characteristic properties and particularly their ability to reversibly tailor their mechanical properties (0.1–100 kPa) by varying mainly the chemical composition^4,5^. Also, they can be dynamically stimulated by a variety of stimuli, such as electric field, pH, and light, and thus provide excellent prospects for controlling the dynamic extracellular environment that cells feel *in vivo*^6,7^.

Polyacrylamide (PAm) has been one of the most exploited gels for studying the mechanotransduction of cardiomyocytes^8^ because it is easy to make and control in stiffness. NRVMs maturation on these materials has shown a significant dependency on the hydrogel stiffness^9^. Cells on soft PAm substrates (1–50 kPa) exhibit a mature phenotype, a well-organised cytoskeleton and a reasonable excitation threshold^9,10^. The magnitude of calcium stored in the sarcoplasmic reticulum and the expression of the sarcomeric calcium pump (SERCA2) were found to be greater on substrates with the elastic modulus of 10 kPa (calcium storage of almost 0.08 mM) in comparison to cells seeded on 1 kPa substrates^9^ (calcium storage of almost 0.03 mM). Topography has a large influence on cell alignment; cardiac cells are sensitive to both nano- and micro-scale topographies^11,12^. Heidi Au *et al*. have demonstrated that cell alignment was greater on 0.5 µm deep grooves than 3 µm deep grooves^13^. Other studies demonstrated an improving into the alignment of cell-seeded on patterned polyethylene glycol (PEG) hydrogels (800 nm width grooves and ridges) in comparison to unpatterned constructs^14^.

Nevertheless, hydrogels have interesting properties for application in cell culturing, cells typically adhere poorly to them, and the functionalization with adhesion proteins is necessary^15^. Patterning synthetic hydrogels are not straightforward. In the particular case of PAm, two main techniques are generally employed for patterning biomolecules: (1) covalently attaching proteins on activated regions^5^ and (2) copolymerizing of ECM proteins directly onto the gel^16^ via microcontact printing (µCP). The latter involves the preparation of an elastomeric stamp generally made of polydimethylsiloxane (PDMS) that is directly printed atop of PAm pre-polymer solution. Di Benedetto *et al*.^17^ have demonstrated that this technique is possible to transfer the anisotropic pattern on PAm and have an anisotropic replica with grooves and ridges wide from 2 to 100 µm. However, compared to PDMS, Parylene C is typically preferred for patterning PAm hydrogels for two main reasons. Firstly, Parylene C mould preparation does not require complicated microfabrication steps. Secondly, Parylene C has a lower oxygen permeability than PDMS^18^, and this allows an efficient preparation of PAm, whose polymerisation is inhibited by the presence of free oxygen radicals. A photoresist lift-off patterning method was also recently reported for patterning biomolecules on PAm with high fidelity^15^. Moreover, while this technique allows for the control of the shape and size of epithelial cells, it remains as non-trivial.

In that direction, we recently proposed a simple and versatile method for patterning PAm hydrogel scaffolds either topographically and biochemically using Parylene C masks^19^. Herein, we leverage this approach and demonstrate its use in NRVM culturing. We particularly focus on studying cell elongation, morphology, and orientation on flat PAm uniformly-covered with collagen (FPC) and grooved collagen-patterned PAm (GPC). Overall, we demonstrate that the patterns on GPC scaffolds promote significantly cellular alignment, while an increase of the calcium activity is also recorded.

## Materials and Methods

### Collagen IV preparation

5 mg of type IV human placenta collagen (Sigma-Aldrich, C7521) was reconstituted in 5 mL of sterile 1 X phosphate buffered saline (PBS) and let mix on a roller shaker for 3 hours. The solution was then split into five aliquots of 1 mL and stored at −20 °C.

### Copolymerization of collagen IV on a polyacrylamide gel

Flat polyacrylamide covered with collagen (FPC) and grooved collagen-patterned polyacrylamide (GPC) were compared to demonstrate that the technique showed in a previous work^19^ can be applied to mimic in the vitro cardiac extracellular environment and promote NRVM alignment. In this work, we describe only the fabrication of FPC since the fabrication of GPC was explained in^19^. The spacing value of GPC (width of the ridges and grooves and depth of the grooves) was chosen as the optimum pattern for NRVMs based on the literature (i.e., 10 µm width and 1 µm deep grooves)^20^.

**Figure 1:**
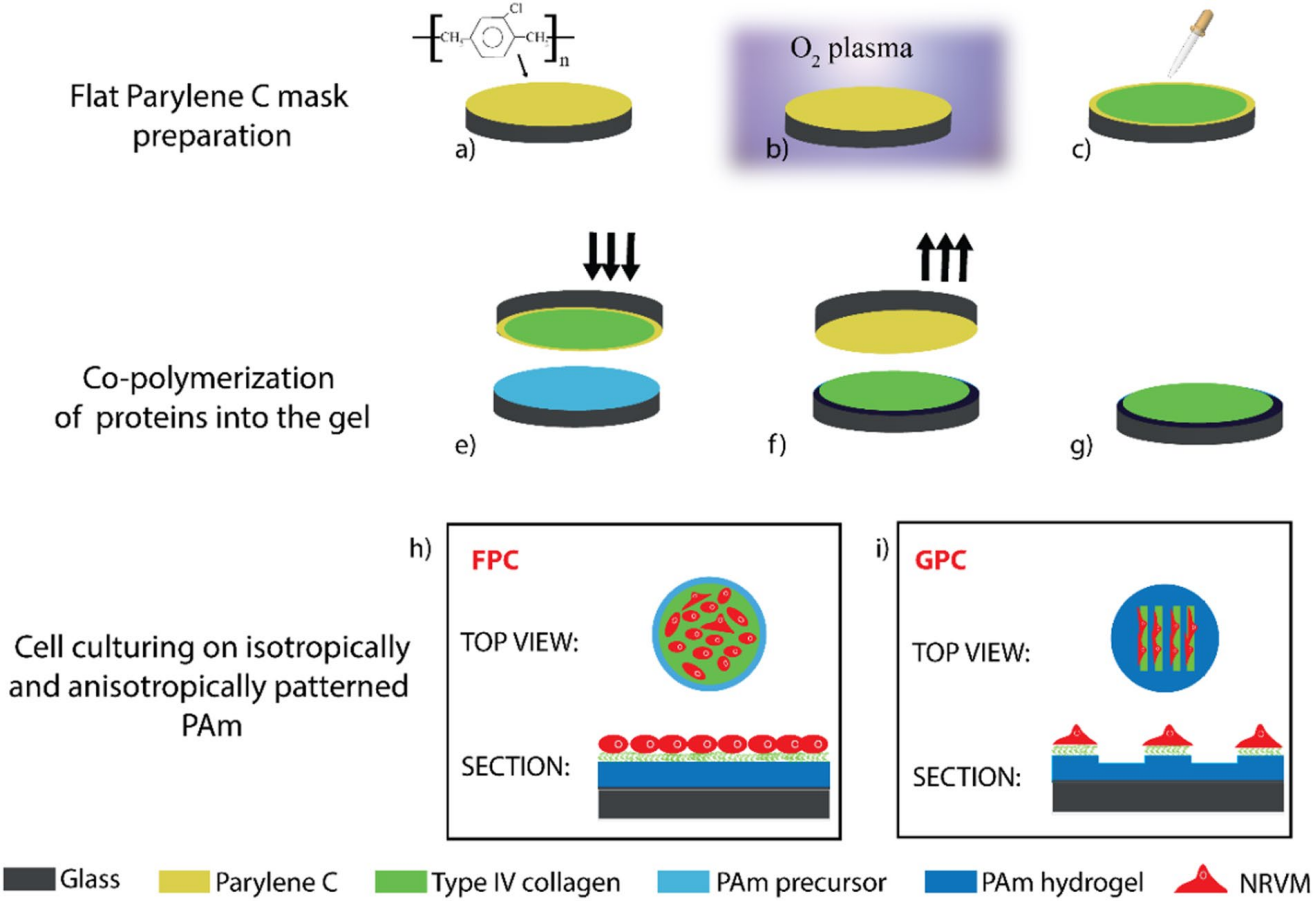
Sketch of the functionalization of flat polyacrylamide hydrogel with collagen (FPC) and cell culture application. Parylene C is placed on glass coverslips (**a**), treated with oxygen plasma (**b**) and uniformly coated with collagen (**c**). PAm pre-polymer solution is gently pipetted on activated glass coverslips and Parylene C mask is put in contact with the solution (**e**). After hydrogel polymerisation, the mask is removed (**f**), and FPC samples are stored in PBS (**g**). In (**h**) and (**i**) top and section views of FPC and GPC are shown with cells randomly distributed in FPC and aligned in GPC.

Briefly, this method relies on the copolymerization of collagen IV into the gel during the direct contact of the acrylamide precursor mix with a protein-activated Parylene C films. Glass coverslips (13 mm in diameter) were washed in acetone, isopropanol, and deionised water and then dried using nitrogen gun. Parylene C films (6–7 µm thick) were then deposited on the coverslips (Fig. 1a) by chemical vapour deposition using a commercially available parylene coater (PDS2010). The silane 3-trimethoxysilylpropyl methacrylate (A174) was used to improve the adhesion of the Parylene C film on the glass. Samples were treated with O_2_ plasma for 1 min and 40 s using an inductively coupled plasma reactor (ICP, OPT 100 ICP 380, Oxford Instruments Plasma Technology) (Fig. 1b). Plasma was generated at a pressure of 1.33 Pa, flow 100 sccm, 1000 W source power, and 20 W bias generator power. The time of exposure to plasma was identical to the one used for preparing the patterned Parylene C masks with hydrophobic/hydrophilic regions^19^. 10 µl of protein solution was then spread on the activated Parylene C films and masks were let dry under a fume hood for 30 min (Fig. 1c).

5 mL polyacrylamide hydrogel precursor with a total polymer content of 8.48% (w/v) and crosslinker concentration of 5.66% (w/w) was prepared. The solution was degassed for 1 h to remove all the dissolved gas that could limit the free radical polymerisation. To initiate gelation, 3 μL of 10% w/v ammonium persulfate (APS, Sigma-Aldrich, A3678) was added to 300 μL of gel precursor solution followed by 0.3 μL of N, N, N′, N′-Tetramethylethylenediamine accelerator (TEMED, Sigma-Aldrich, T9281). A 60 μL of gel precursor mix was gently pipetted on activated glass coverslips^5^ and sandwiched with protein-functionalized Parylene C masks (Fig. 1e). Gels were then left to polymerise at room temperature for one h (Fig. 1f). After polymerisation, masks were then removed and the gels were stored in PBS at 4 °C (Fig. 1g). Constructs were directly transferred in sterile tissue culture petri dish covered with cell culturing medium (Fig. 1h,i).

### Contact angle measurements

Parylene C (6–7 µm) was placed as described previously on glass coverslips of 13 mm of diameter. PDMS was instead spin coated at 6000 rpm (~ 8 µm) on glass coverslip of 13 mm of diameter and cured in an oven at 75 °C for 1 hour. Sessile deionised water 10 µl droplets were gradually engaged on the surface of Parylene C and PDMS to measure the static contact angle. A Drop Shape Analysis System (DSA 30 Kruss Co., Germany) was employed for the calculation of the contact angle. A polynomial function was fitted to the two-3-phase sections of the profile in the region of the baseline. One measurement per three samples per each category was performed, and the average values were extrapolated. Samples were blown with nitrogen gun before each measurement. Samples were stored at room temperature (22 °C) and low humidity level (35%).

### Topography characterisation

The topography of flat polyacrylamide was visualised using environmental variable-pressure (VP) scanning electron microscopy (SEM) (Zeiss EVO 50XVP). Almost 100 µm thick PAm was fixed on glass coverslips as described above and it was hydrated overnight in deionised water. Samples were speckled gently with Kimwipe and loaded atop SEM stubs using carbon tape. VPSE detector at 20 kV acceleration voltage was used. Measurements were taken in a reasonable time to preserve the gradual loss of water of the gel over time. The topography of Parylene C mask and PAm was evaluated using a stylus profiler (KLA-Tencor) and by using an optical microscope (Zeiss Axio Lab) at 50x magnification. Three measurements on three samples were taken for the statistical analysis.

### Mechanical characterisation at the nano-scale

Flat polyacrylamide samples were prepared using the polymerisation between two activated coverslips as described elsewhere^5^. They were stored either in PBS or water at 4 °C before the nanoindentation tests. Our experimental setup for measuring the mechanical properties is a hybrid system made of a commercial head (SMENA, NT-MDT) with home-built electronics. Hydrogels were tested both in water and in PBS.

A spherical tip of 5 µm of diameter (sQUBE, CP-CONT-BSG-A) was used to minimise the indentation. Cantilever stiffness calibration was done following the work of Sader *et al*.^21^, and it was estimated to be 0.2 N/m with an error of 10%. Samples were placed in a petri dish of 35 mm of diameter filled with liquid. The approaching/retraction rate used was 400 nm/s.

Since the hydrogels showed a negligible adhesion, we have analysed the force-distance curves using the Hertz model. Young’s modulus can be thus deduced from the equation 1 as follows^22^:

1 $${F}_{N}=\frac{4E\sqrt{R{\delta }^{3}}}{3(1-{v}^{2})}$$

where F_N_ is the maximum force applied, E is Young’s modulus, R is the radius of the spherical tip, the indentation depth is δ = (z − d) where z is the piezo position and d is the cantilever deflection. ν is the Poisson’s ratio equal to 0.48^23^.

### NRVM isolation and culture

The study with NRVMs was exempted from formal ethics review. NRVMs were isolated from Sprague-Dawley rats two days after birth in compliance with Schedule 1 methods (Scientific Procedures) Act 1986. The isolation technique has been described previously^11,24^. Briefly, hexempteart ventricles were minced and enzymatically digested. After the digestion, the tissue was centrifuged to produce a cell suspension which was filtered to remove any non-digested tissue. Cells were then suspended again in 25 ml NRVM medium (67% Dulbecco’s modified Eagle medium (DMEM), 16% Medium 199, 10% Horse serum (Gibco), 4% foetal bovine serum (FBS) (Gibco), 2% HEPES (4-(2-hydroxyethyl)-1- piperazineethanesulfonic acid) buffer and 1% penicillin-streptomycin). The suspension was then plated in T75 flasks for 1 hour at 37 °C to remove fibroblasts. NRVMs were collected and counted using a hemocytometer. NRVMs were plated on FPC and GPC substrates, and they were successively incubated (37 °C, 5% CO2). The NRVM medium was replaced every 2–3 days. All experiments were performed 3–4 days post seeding.

### Immunostaining

Monoclonal anti-*α*-actinin antibody (Sigma, A7811), deoxyribonucleic acid labeled with fluorescent 4′,6-diamidino-2-phenylindole (DAPI) (Invitrogen) staining were used to quantify cell alignment. Fluorescent images were obtained using an inverted Zeiss LSM-780 confocal microscope with x40 oil-immersion lens (Carl Zeiss).

### Quantification of cellular alignment, morphology, and orientation

Each cell was approximately fitted with an ellipse whose minor (D_min_) and the major axis (D_max_) was defined as the length and width of individual myocyte. D_max_ of each cell was selected relative to the horizontal axis of the image field. Elongation was described through the elongation factor (D_max_/D_min_ − 1) that describes the extent of the equimomental ellipse lengthened or stretched out^25^. The values of the elongation factor were determined from each scaffold on almost n = 15 cells randomly selected from each sample.

Cell area and circularity were analysed to study cell morphology using dedicated software, ImageJ (http://rsb.info.nih.gov/ij). Circularity is defined in the equation 2 ^26^:

2 $$Circularity=\frac{4\pi A}{{P}^{2}}$$

where P is the perimeter and A is the cell area, quantified using ImageJ. Circularity index equal to 1 indicates a non-elongated cell.

The cell orientation angle (θ) was measured manually using an ImageJ software package (http://rsb.info.nih.gov/ij) as the lack of deviation between the major elliptical axis and the reference axis of a single cell. For alignment of cells on grooved substrates, the direction of the pattern (preferential mean axis) was considered as a reference axis, whereas for alignment of cells on the flat substrates an arbitrary axis of alignment was chosen. The minimum alignment value close to 10° denotes parallel alignment with the direction of the pattern, and the maximum value close to 90° represents perpendicular alignment. Cells with an orientation angle of less than 10° were counted as aligned cells^27^. Orientation angles are reported in polar plots obtained using ready-made routines in MATLAB.

Nuclei orientation was quantified as described previously via conversion of the DAPI channel images into binary images using ImageJ^11^ to recognise any ellipse (nuclei) present with the size set to 10–50 µm. Alignment was defined as the lack of deviation in the axis of an individual nucleus from the mean axis of all individual nuclei^11^. The ellipses were only counted if they were between the set range to exclude non-nuclei or composite structures from the analysis. At least 90 cells were randomly selected per each category of scaffolds to determine cell shape and orientation. Each analysis was performed on fluorescence images (three images per each scaffold) at the same magnification.

### Ca^2+^ transient measurements

Fluo-4-acetoxymethyl ester (fluo-4 AM) (Invitrogen, ThermoFisher Scientific) was used to visualise the intracellular calcium transients. NRVMs were loaded with 4 μL fluo-4 AM (prepared as 50 µg Fluo-4 AM dissolved in 50 µL DMSO) in 1 mL DMEM and placed in the incubator at 37 °C for 20 min^11,20^. The DMEM was refreshed, and the cells were returned to the incubator for a further 20 minutes for de-esterification of the dye. The constructs were mounted on the stage of an upright Nikon Eclipse FN1 microscope or an inverted Nikon Eclipse TE2000 microscope in a glass bottom dish (MatTek Corporation) and observed through a 40x water immersion or 40x oil objective respectively. Ca^2^ transients in cardiomyocytes were studied by field stimulating the cells at 1 Hz to induce rhythmic depolarisation, and line scans were recording. A custom made MATLAB code was used to calculate the normalized amplitude as f/f0, time to peak (Tp), times to 50% (T50) and 90% declines (T90) in the transients^28^.

### Statistical analysis

All the described experiments were repeated at least three times. With regards to topography, nonparametric analysis was carried out among samples to statistically evaluate the significant difference between ridges and grooves of the mask and the gel, respectively. A Mann-Whitney test was also performed to compare the two groups, and the significance was fixed at 1%.

For statistical analysis of cell elongation, morphology and orientation, three different images were acquired per each sample to analyse at least 80 cells for statistical analysis.

Mann-Whitney test was employed for evaluating cell elongation, circularity, and area whereas nuclei alignment and calcium transient were performed using an unpaired t-test. Cell area and circularity are presented as box plots, and each box is composed of whiskers indicating the lower extreme and upper extreme of the data, while the top part and the bottom part of the box are the first and third quartiles. The line inside the box is the median. In the plots, *indicates p < 0.05 and **indicates p < 0.01. The statistical analysis and graphic presentation were performed using OriginPro v.8 SR2 software and Prism 7 software (GraphPad Software Inc.). All data are expressed as a mean ± standard error of the mean.

### Data Availability

The data that support the findings of this study are available from the University of Southampton institutional repository at: https://doi.org/10.5258/SOTON/D0605.

## Results and Discussion

### Hydrogel patterning and characterisation

**Figure 2:**
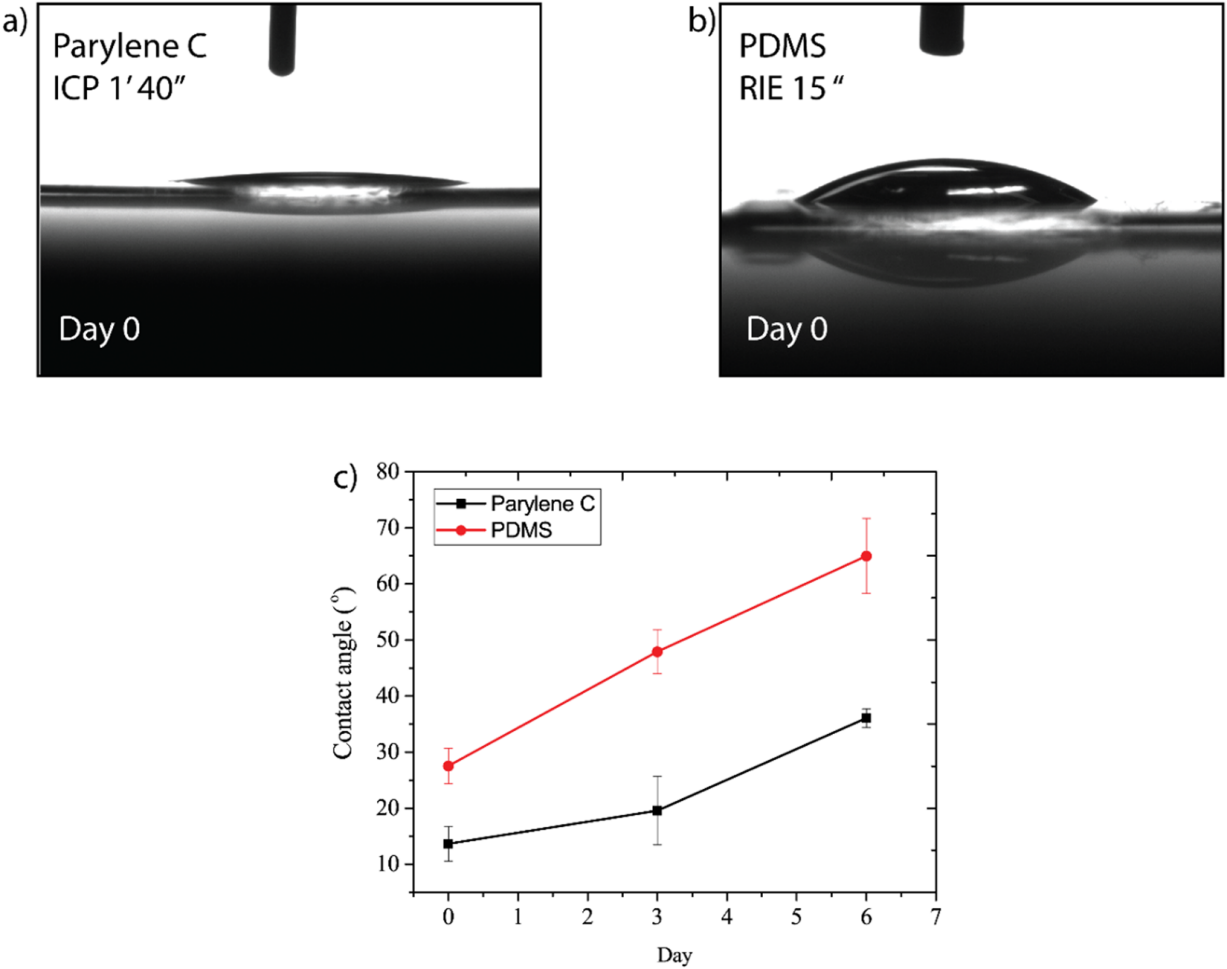
Contact angle measurements. In (**a**) and (**b**), images of 10 µl DI water droplets on Parylene C and PDMS at day 0, immediately after the plasma treatment. In (**c**) the graph reports contact angle measurements as a function of days.

The anisotropic pattern on PAm was obtained using capillary-based approach^17^ thanks to capillarity of the pre-polymer solution in hydrophilic vertical channels created in Parylene C masks. Parylene C after plasma oxygen restores its hydrophilicity slower than PDMS^29^. The feature height on the hydrogel strongly depends on the contact angle, the hydraulic radius of the capillary and the viscosity of the pre-polymeric solution^17^. We have monitored the contact angle of flat Parylene C and PDMS with the same thickness (~7 µm) using ICP and reactive ionic etching (RIE). ICP treatment was used to hydrophilize Parylene C for 1 minute and 40 seconds and RIE was used to treat PDMS with oxygen for 15 sec. The time of exposure and the kind of treatment were decided based on the fabrication process that has been employed for the two materials. Figure 2a,b show 10 µl water drop on Parylene C and PDMS immediately after plasma treatment. The contact angle of Parylene C and PDMS before plasma oxygen are 84.2 ± 7.6° is 117.4 ± 2.8° respectively. Parylene C restore its hydrophobicity slower than PDMS^29,30^, and we found that after 6 days the contact angle for Parylene C and PDMS was 36.07 ± 1.66° and 64.97 ± 6.72° respectively. In Fig. 2c the measurements of the contact angle for six days are shown.

**Figure 3:**
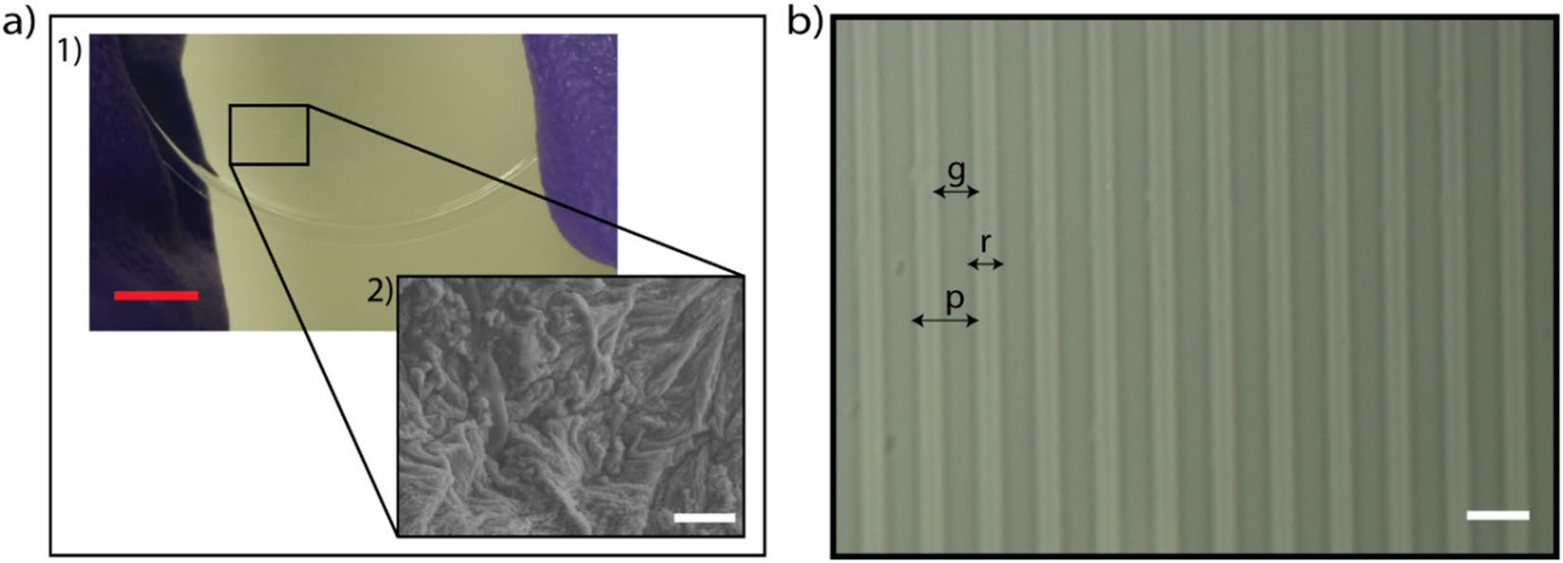
Surface VP SEM photograph of flat hydrogel surface and an optical micrograph of the patterned hydrogel. The image (**a.1**), shows a hydrogel fixed on the glass coverslip and (**a.2**) is a representative SEM image of flat polyacrylamide. In (**b**) is shown a micrograph (50x objective) of grooved PAm characterised by pitches (p), ridges (r) and grooves (g) after removing the Parylene C mask. (Red scale bar: 1 cm. White scale bar: 20 µm).

The surface of flat polyacrylamide was obtained avoiding surface aberration, and the native hydrate state of the gel was preserved^31^. In this work, we have used PAm fixed on glass coverslip with a defined formulation to fix the structure and the elasticity of the material^5^. PAm SEM images generally present pores and voids on the surface because of the failure of the structures during preparation for imaging (e.g., cryo-freeze-dry) and the volume fraction and the distribution of polymeric chains can change accordingly^32^. The hydrogels were fixed on glass coverslips obtaining a uniform film of almost 300 µm of thickness (Fig. 3a.1). The topography of flat PAm prepared with T = 8.48% (w/v), and C = 5.66% (w/w) was checked using SEM as illustrated in Fig. 3a.2, and it results more wrinkled and jagged without evident porosity since the gel was checked immediately after removing it from water and it was almost hydrated.

The comparison between the topography of the mask and the hydrogel after patterning was also evaluated extending the results already reported in our previous work^19^. Grooves (g), ridges (r) and pitches (p) of the hydrogels in the hydrated state are illustrated in Fig. 3b. The average values of g, r, and p of the masks were here analysed using the profiler, and they were 6.88 ± 0.6 µm, 6.32 ± 0.1 µm and 20.62 ± 0.1 µm respectively. The average height of the grooves of the mask was found to be 0.99 ± 0.04 µm.

The averaged values of the width of g, r and p of the hydrogels were found to be10.1 ± 1.05 µm, 7.8 ± 1.24 µm and 19.7 ± 1.28 µm respectively. The comparison between the ridges of the mask and the grooves of the gels was not significant with p > 0.01. The average height of the grooves of the hydrated hydrogels was evaluated using the profiler (low force of ~1.96 mN), and it was almost 1 µm, close to the height of the grooves of the mask.

### Elastic modulus values

**Figure 4:**
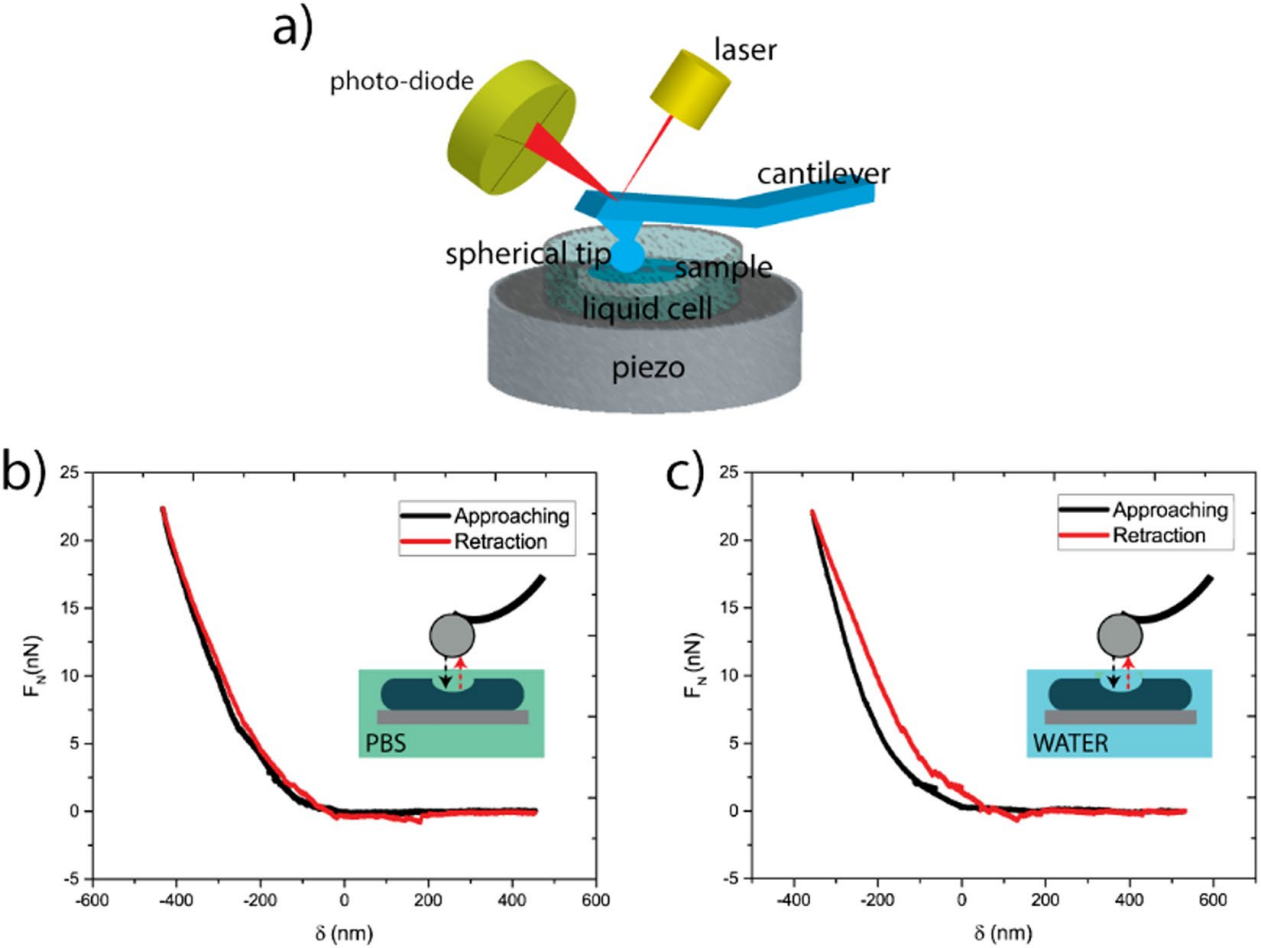
Elastic modulus evaluation. The draw of AFM indentation in liquid (**a**) and force-indentation curves (**b**,**c**) obtained from flat PAm samples underwater (**a**) and PBS (**b**).

Nanoindentation tests were performed on flat PAm samples as illustrated in Fig. 4a. These tests were necessary to quantify Young’s modulus of the substrates, important parameters for cell elongation and function^5^. They cannot be carried out on the microstructured ones since the radius of the AFM tip was comparable with the width of the grooves. As already mentioned sharper tips would induce plastic deformation on such soft materials. In any case, the contact area between an AFM tip and a steep ridge can never be defined due to the geometry of the system.

Typical load-displacement curves for PAm show a region of the negative load in the unloading portion due to the adhesion between tip and sample (Fig. 4b,c). Measurements under water and PBS present negligible adhesion force values. Both curves do not present jump to contact and show a linear elastic behaviour. Thus in both cases, the Hertzian model can be applied. The E value was found to be 37.1 kPa for PAm measured in water, and 22.9 kPa for PAm measured in PBS. The difference might be due to local changes in the gelification process. However, the two values fall in the desired range for our application.

### Cell morphology and alignment

In this work, we have applied a method previously reported to functionalize polyacrylamide hydrogel with collagen IV using Parylene C masks^19^. Polyacrylamide without collagen treatment was cytotoxic for NRVMs as reported in Fig. S1 of Supplementary Information. As expected, cells on substrates without adhesion protein display an apoptotic behavior because of non-adhesiveness properties of PAm hydrogel^15^. Therefore, adhesion protein is necessary for improving cell adhesion. With our proposed technique we have previously demonstrated that protein is completely transferred from selectively hydrophilized Parylene C mask onto the hydrogel^19^. In this way, we can obtain a more biocompatible polyacrylamide based scaffolds using a simple and non-toxic technique. In this paper, we supplement our previous findings with the application of this technique towards NRVM culturing.

**Figure 5:**
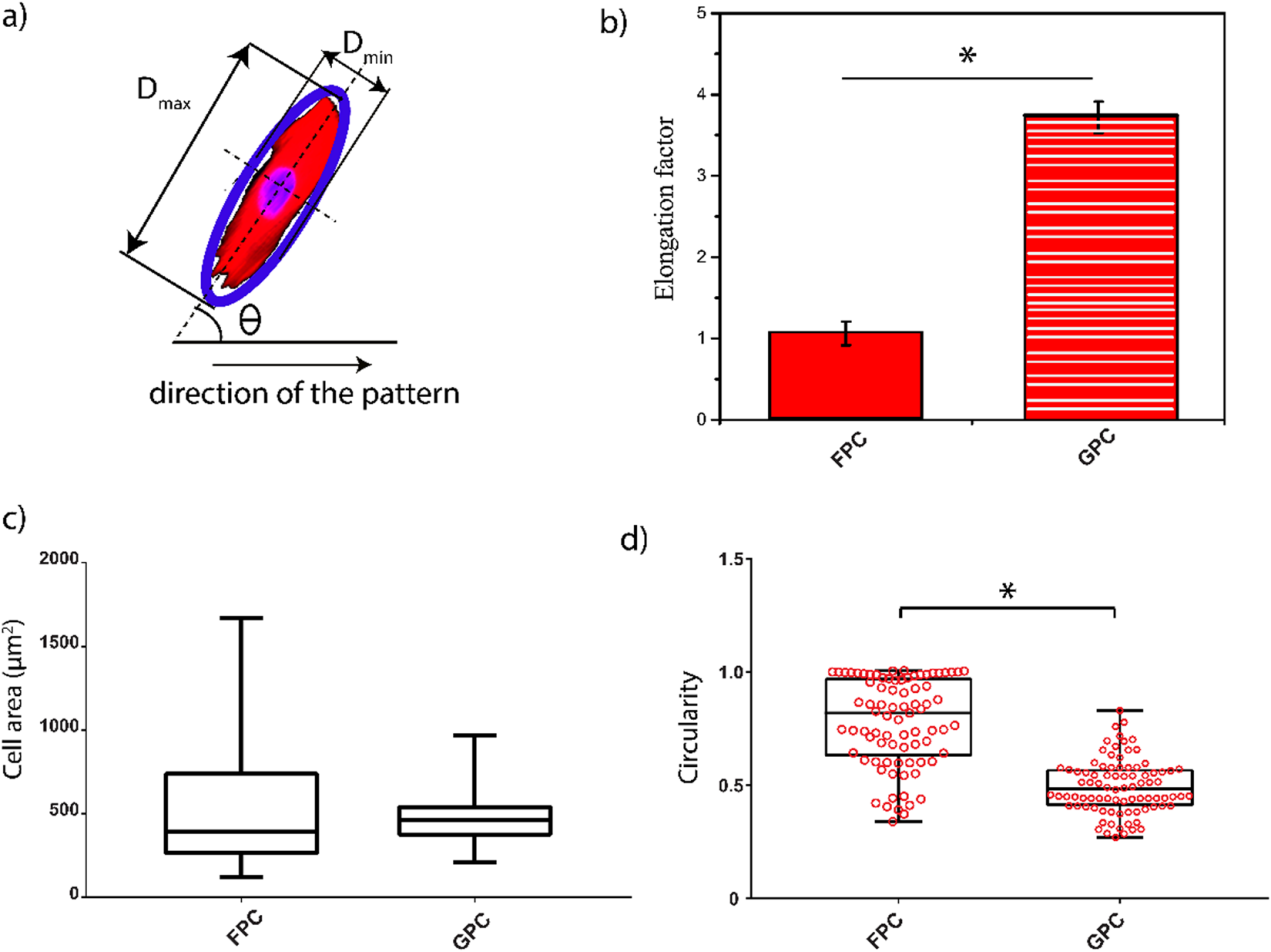
Cell morphometric analysis. Sketch of cell morphology and orientation along the collagen stripe (**a**). D_max_ and D_min_ are the long and short axis, θ the angle of orientation of the cells. Cell elongation is quantitatively evaluated through the elongation factor (**b**). A significant elongation between the two types of samples is evident in (**b**). Box plots of cell areas (**c**) and scatter box plots of circularity distributions (**d**) are shown in the bottom panel. A significant difference was found in cell circularity between cells seeded on FPC and GPC. Notes: *p < 0.005, each box has its ends at the quartiles, and the band within the boxes is the median of the distribution ((**c**), (**d**)).

PAm hydrogels are widely used as a scaffold in cell biology especially for cardiomyocytes since it has mechanical properties close to the extracellular matrix of the heart. However, most of the techniques used to pattern this hydrogel are toxic and not easy to carry out^16,33^. For this reason, we considered the application of this technique for cardiac tissue engineering creating biomimetic platforms that can be applied in synthetic biology. The comparison between isotropic and anisotropic substrates is made to demonstrate the effects of these platforms on cell morphology, orientation, and activity. For each cell, a best-fitted ellipse was outlined with a minor and major axis. A certain orientation (θ) of the major axis concerning the reference axis was considered as shown in Fig. 5a.

Cellular alignment plays an important role in functional and physical characteristics of many tissues, especially for the heart. To demonstrate the application of our substrates on cell elongation quantitative data were extracted. The elongation factor is commonly used to quantify cellular alignment in tissue engineering. Generally, this parameter assumes 0 value for a perfect circle and 1 for an ellipse with an axis ratio of 1:2^34^. The results shown in Fig. 5b demonstrate the efficiency of GPC scaffolds in promoting the elongation of NRVMs. The elongation factor is significantly higher for cells seeded on GPC with an averaged value of 3.75 ± 1.49 in comparison to cells seeded on FPC that have a lower value (0.70 ± 0.67). These results prove that collagen-patterns have a significant influence on NRVMs behaviour.

Cell area and circularity quantified cell morphology. Figure 5c shows the distribution of cell areas on FPC and GPC. Cell areas on FPC are more positively skewed with more dispersed values. The average area of cells on FPC was found to be 558.04 ± 428.33 µm^2^ in comparison to the area of cells seeded on GPC (average area: 480.04 ± 160.01 µm^2^). The evaluation of the cell circularity is reported in Fig. 5d. Cells on FPC were more circular as presented in a whisker plot where data are negatively skewed with an average value of 0.85 ± 0.14. Cells on GPC present a less circular morphology, and data dispersion is narrower and slightly positively skewed with an average value of 0.50 ± 0.12. The distributions of circularity denote a significant decrease when cells are seeded on patterned substrates (p < 0.005).

**Figure 6:**
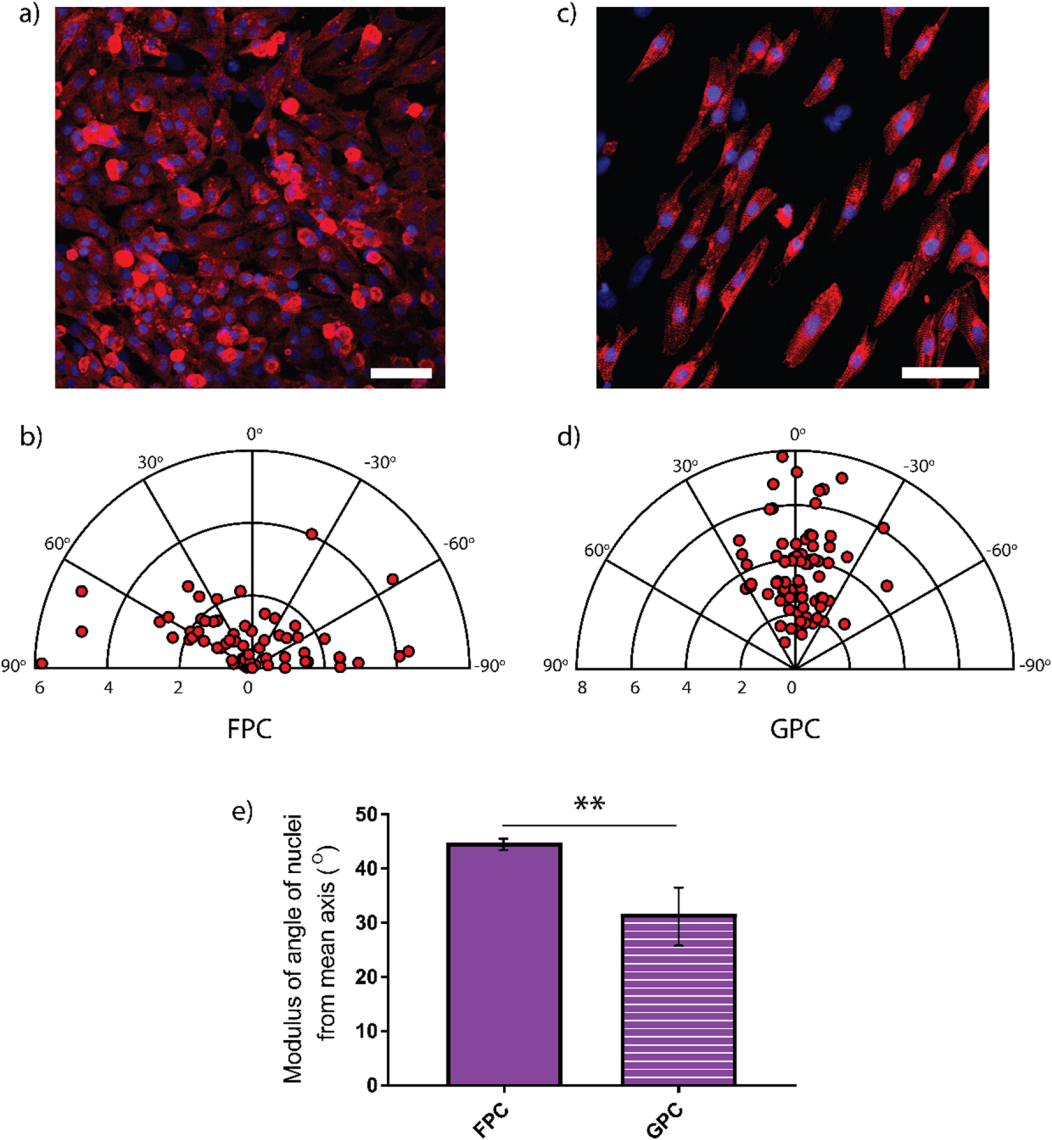
Immunofluorescent staining images, analysis of cell and nuclei alignment on FPC and GPC. Representative immunofluorescence staining of sarcomeric α-actinin (red) and nuclei stained with DAPI (blue) on FPC (**a**) and GPC (**c**). Relative polar plots of the orientation angle of cells on FPC (**c**) and GPC (**d**) are also reported. In this polar plot the distance from the origin, R, is the elongation factor, each point is a single cell (81 cells for FPC and 85 cells for GPC) and the angle to the horizontal, θ, is the angle the long axis of the cell makes with the reference axis. In (**e**) is shown the modulus of the angle of nuclei alignment on uniformly covered and collagen-patterned polyacrylamide with **p < 0.01.

Cellular orientation alongside the direction of the anisotropic pattern was evident from optical images. We confirmed and quantified the orientation of cells using polar plots to show the difference in orientation of NRVMs on the two type of samples. In the polar plots, the azimuth ranges from −90° to 90°, and the 0° corresponds to the elongation direction of the pattern. A perfectly circular cell would be at the origin, and long and thin cells are far from the origin. Cells on FPC showed a random distribution (Fig. 6a) with more dispersed values close to the origin of the graph as reported in the graph of Fig. 6b, whereas cells on GPC have the preferential orientation alongside the pattern (Fig. 6c). In the polar plot of Fig. 6d, there are not points close to the origin, confirming that cells on GPC are more elongate than cells cultured on flat uniformly collagen-patterned scaffolds. The percentage of cells with angle values 15° < θ < 15° on FPC is 8.64% less than on GPC where it was found to be 77.64%. Also, cells with random distribution in uniform samples are 50.6° ± 23.4°. On collagen-patterned surfaces, the values of angles are 11.69.2° ± 9.9°.

The modulus of the angle of nuclei was also measured using DAPI staining. Results reveal that the modulus of the angle of nuclei from the mean axis is smaller for cells seeded on GPC (31.70° ± 4.16°) than cells seeded on FPC (44.75° ± 0.81°) with a considerable alignment to the direction of the pattern as shown by the histogram in Fig. 6e.

**Figure 7:**
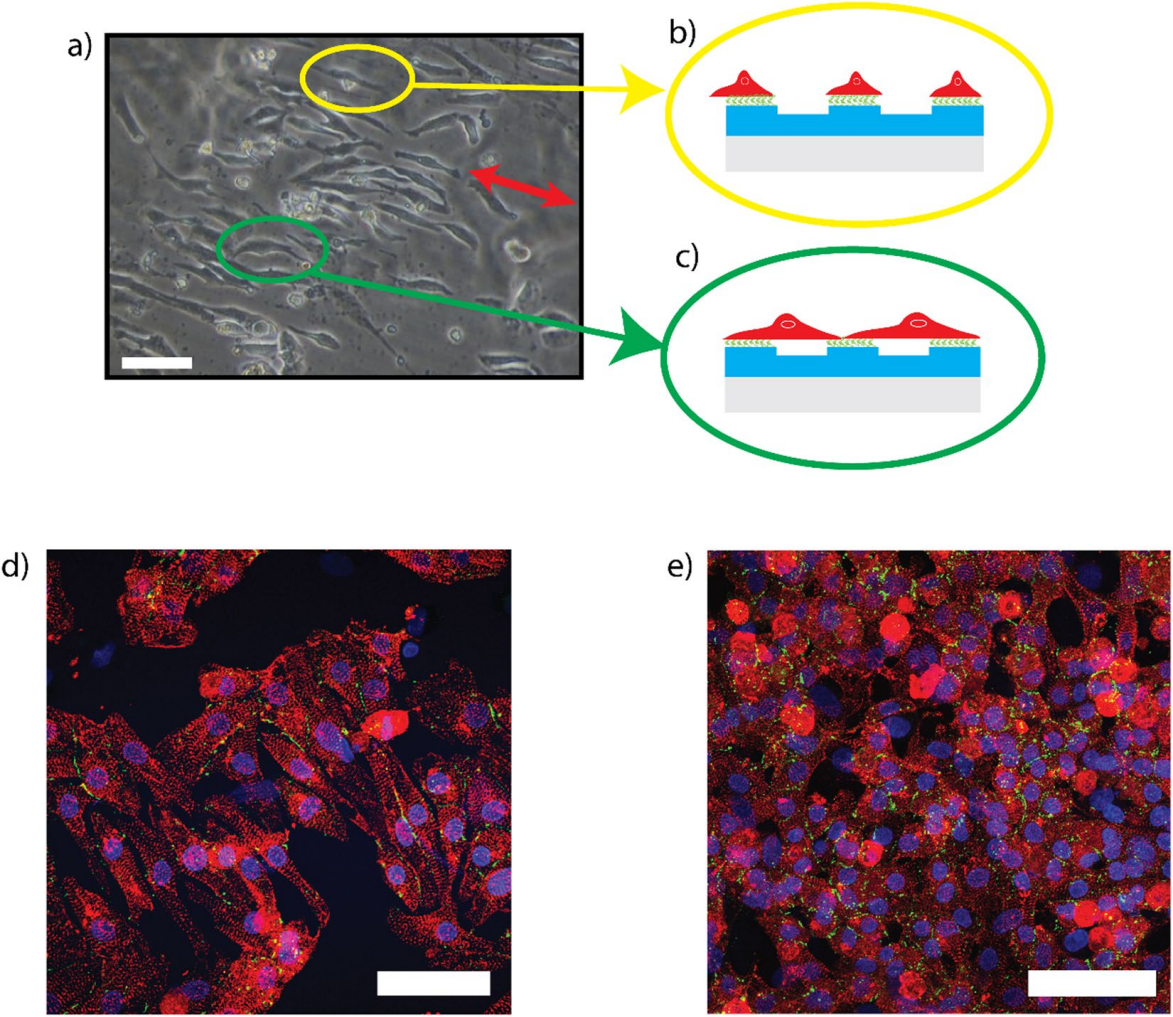
Cell tendency to bridge across the anisotropic pattern. Bright field image of NRVMs aligned on GPC (**a**) and a sketch of cells aligned alongside the features (yellow circle, (**b**)) and bridges across the lines (green circle, (**c**)). Immunofluorescence images of cells aligned on GPC and glass control with an expression of connexin43 (green) are shown in (**d**) and (**e**) respectively. (White scale bar: 50 µm).

The bright field image in Fig. 7a shows the direction of the lines (red arrow) and also the considerable tendency of the cells to follow the pattern. With these results, we also demonstrate that cells alignment is strongly aimed by cell-ECM interaction so effectively that cells follow the ridges functionalized with collagen (Fig. 7b) or the bridge across the ridges (Fig. 7c) to create focal adhesions. Immunostaining for connexin43 (Cx43) showed that gap junctions in sarcomeric α-actinin were aligned along the perimeter of the elongated cardiomyocytes cultured on GPC, resulting in spatial distribution of Cx43 primarily oriented parallel to ridges (Fig. 7d), versus more random distribution in glass control (Fig. 7e). The cx43 expression on cell aligned is indicative of well-formed intracellular contact^35,36^. In our case, Cx43 also shows random membrane distribution typical of neonatal cardiomyocytes^36^.

### Cell electrophysiology

**Figure 8:**
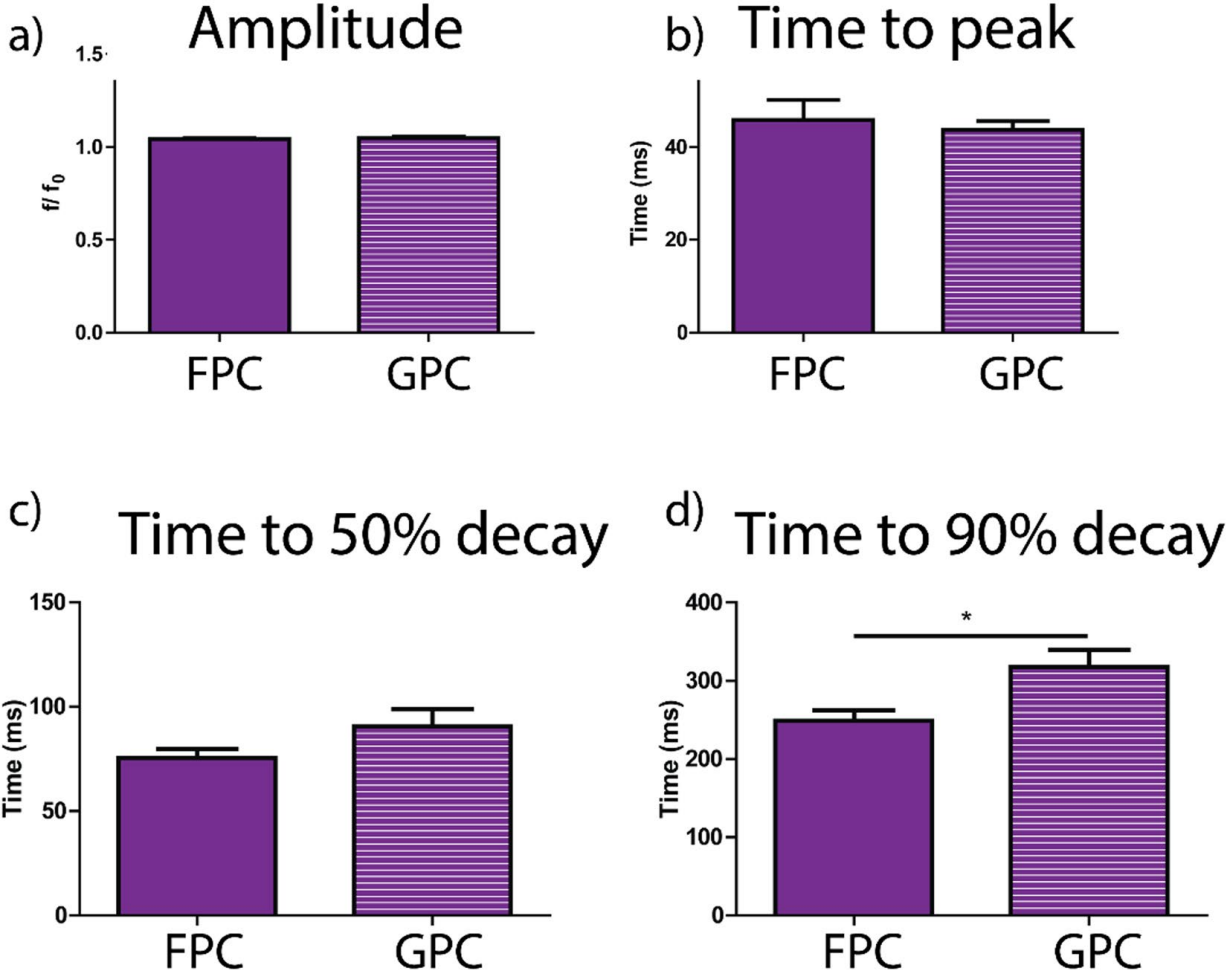
Ca^2+^ transient amplitude (f/f0), time to peak (Tp), 50% time to decay (T50) and 90% time to decay (T90) of NRVM cultured on unpatterned (FPC) and patterned (GPC) substrates. In (**a**) Ca^2+^ signal is shown as f/f0 with fluorescence intensity, f, normalised to the minimal intensity, f0, measured between 1 Hz contractions. In (**b**) time to peak of Ca^2+^ release in FPC and GPC. In (**c**) and (**d**) transient decay times (T50 and T90) are shown, *p < 0.05.

Different patterning techniques have been employed to improve Ca^2+^ cycling in NRVMs^11,24^. It has been shown that microgrooved substrates can increase the diastolic and systolic Ca^2+^ dynamic properties^37^. Many studies have also revealed that the substrate stiffness can influence the electrophysiology activity of the cardiomyocytes^38^. In this work Ca^2+^ cycling properties of NRVMs was studied fixing the stiffness of the scaffolds and comparing two groups: unpatterned (FPC) and patterned (GPC) constructs. No significant differences were found in the amplitude (f/f0), time to peak (Tp) and time to 50% transient decay (T50) between cells cultured on flat and grooved substrates (Fig. 8a–c), but there is only a significant increase for the 90% time decay (T90) with p < 0.05 (Fig. 8d). We have shown that fixing the stiffness and improving cell alignment Ca^2+^ amplitude remains the same (1.03 ± 0.02 in FPC and 1.04 ± 0.02 in GPC), but Ca^2+^ diastolic decay slightly increases on GPC constructs. Since the amplitude in NRVM is the same, there are no significant changes in the sarcoplasmic reticulum (SR) function^37^ and the calcium releasing through ryanodine receptors (RyR)^39^. The amplitude and the time to peak can also be attributed to the peak force generated by the cells on the substrates^8^. Since the stiffness of the substrates has been fixed, cells generate the same force on the substrates with Tp equal to 45 ± 15.5 ms for FPC and 45.12 ± 5.9 ms for GPC. The significant decrease of T90 for cells plated on GPC samples (317.98 ± 61 ms) instead suggests that there is an increase in diastolic Ca^2+^ levels^40^. Slower calcium transients can augment the contraction as myofilaments are exposed to Ca^2+^ for a longer period^41^.

## Conclusions

Mimicking the native extracellular matrix of the cardiac cells is one of the most important steps to design *in vitro* models. The goal of this research was to demonstrate the possible application of new polyacrylamide patterned scaffolds via Parylene C masks. We have used a versatile and simple method to fabricate flat and grooved constructs transferring collagen directly from hydrophilic Parylene C to PAm. We have demonstrated that Parylene C is a better mask than the classic PDMS and it can be used for better patterning PAm hydrogel. Flat and grooved surfaces were also characterized showing how the pattern is faithfully transferred onto the hydrogel. NRVMs behavior on isotropic and patterned substrates was quantified. Collected data on cell morphology, elongation and orientation confirm that a significant cell elongation is induced on anisotropic architecture mimicking the distribution of cells in the native cardiac tissue. These results, even if preliminary, are promising for creating simple *in vitro* platforms necessary to study the effect of drugs for different cardiac pathologies.

## Electronic supplementary material

Supplementary Information 

## References

[1] Schwan J (2016). Anisotropic engineered heart tissue made from laser-cut decellularized myocardium. Sci. Rep..

[2] Suuronen, E. J. & Ruel, M. Biomaterials for cardiac regeneration*. Spinger* (2015).

[3] Geckil H, Xu F, Zhang X, Moon S, Demirci U (2011). Engineering hydrogels as extracellular matrix mimics. Nanomedicine.

[4] Denisin AK, Pruitt BL (2016). Tuning the Range of Polyacrylamide Gel Stiffness for Mechanobiology Applications. ACS Appl. Mater. Interfaces.

[5] Tse JR, Engler AJ (2010). Preparation of hydrogel substrates with tunable mechanical properties. Curr. Protoc. Cell Biol..

[6] Uto K, Tsui JH, DeForest CA, Kim DH (2017). Dynamically tunable cell culture platforms for tissue engineering and mechanobiology. Prog. Polym. Sci..

[7] Castano aG (2014). Protein patterning on hydrogels by direct microcontact printing: application to cardiac differentiation. Rsc Adv..

[8] Jacot JG, McCulloch AD, Omens JH (2008). Substrate stiffness affects the functional maturation of neonatal rat ventricular myocytes. Biophys. J..

[9] Jacot JG (2010). Cardiac Myocyte Force Dvelpoment during Differentiation and maturation. Ann. N. Y. Acad. Sci..

[10] Bhana B (2010). Influence of substrate stiffness on the phenotype of heart cells. Biotechnol. Bioeng..

[11] Rao C (2013). The effect of microgrooved culture substrates on calcium cycling of cardiac myocytes derived from human induced pluripotent stem cells. Biomaterials.

[12] Kontziampasis D (2015). Effects of Ar and O2 Plasma Etching on Parylene C: Topography versus Surface Chemistry and the Impact on Cell Viability. Plasma Process. Polym..

[13] Heidi AHT, Cui B, Chu ZE, Veres T, Radisic M (2009). Cell culture chips for simultaneous application of topographical and electrical cues enhance phenotype of cardiomyocytes. Lab Chip.

[14] Kim HN (2012). Patterning methods for polymers in cell and tissue engineering. Ann. Biomed. Eng..

[15] Moeller, J. *et al*. Controlling cell shape on hydrogels using lift-off patterning. *bioRxiv* 111195 (2017). 10.1371/journal.pone.0189901 PMC5752030 29298336

[16] Grevesse T, Versaevel M, Circelli G, Desprez S, Gabriele S (2013). A simple route to functionalize polyacrylamide hydrogels for the independent tuning of mechanotransduction cues. Lab Chip.

[17] Benedetto FD, Biasco A, Pisignano D, Cingolani R (2005). Patterning polyacrylamide hydrogels by soft lithography. Nanotechnology.

[18] Oppegard SC, Blake AJ, Williams C, Eddington DT (2010). Precise control over the oxygen conditions within the Boyden chamber using a microfabricated insert. Lab Chip.

[19] Sanzari I (2017). Parylene C topographic micropattern as a template for patterning PDMS and Polyacrylamide hydrogel. Sci. Rep..

[20] Trantidou T, Terracciano CM, Kontziampasis D, Humphrey EJ, Prodromakis T (2015). Biorealistic cardiac cell culture platforms with integrated monitoring of extracellular action potentials. Sci. Rep..

[21] Sader JE, Larson I, Mulvaney P, White LR (1995). Method for the calibration of atomic force microscope cantilevers. Rev. Sci. Instrum..

[22] Mossman, B. T. Cell imaging techniques. Edited by Douglas J. Taatjes. Humana (2013).

[23] Boudou T, Ohayon J, Picart C, Tracqui P (2006). An extended relationship for the characterization of Young’s modulus and Poisson’s ratio of tunable polyacrylamide gels. Biorheology.

[24] Trantidou T (2014). Selective hydrophilic modification of Parylene C films: a new approach to cell micro-patterning for synthetic biology applications. Biofabrication.

[25] Crouch AS, Miller D, Luebke KJ, Hu W (2009). Correlation of anisotropic cell behaviors with topographic aspect ratio. Biomaterials.

[26] Genchi GG, Marino A, Rocca A (2016). Barium titanate nanoparticles promising multitasking vectors in nanomedicine. Nanote.

[27] Ahadian S (2012). Interdigitated array of Pt electrodes for electrical stimulation and engineering of aligned muscle tissue. Lab Chip.

[28] Ibrahim M (2012). Cardiomyocyte Ca2+ handling and structure is regulated by degree and duration of mechanical load variation. J. Cell. Mol. Med..

[29] Trantidou T, Prodromakis T, Toumazou C (2012). Oxygen plasma induced hydrophilicity of Parylene-C thin films. Appl. Surf. Sci..

[30] Ma K, Rivera J, Hirasaki GJ, Biswal SL (2011). Wettability control and patterning of PDMS using UV-ozone and water immersion. J. Colloid Interface Sci..

[31] Joubert, L. Visualization of Hydrogels with Variable-Pressure SEM. **15**, 8–9 (2009).

[32] Trappmann B (2012). Extracellular-matrix tethering regulate stem-cell fate. Nat. Mater..

[33] Tang X, Yakut Ali M, Saif MTA (2012). A novel technique for micro-patterning proteins and cells on polyacrylamide gels. Soft Matter.

[34] Abdullah, C. A. C. *et al*. Aligned, isotropic and patterned carbon nanotube substrates that control the growth and alignment of Chinese hamster ovary cells. *Nanotechnology* **22** (2011). 10.1088/0957-4484/22/20/205102 21444962

[35] Kim D-H (2010). Nanoscale cues regulate the structure and function of macroscopic cardiac tissue constructs. Proc. Natl. Acad. Sci. USA.

[36] Bian W, Jackman CP, Bursac N (2014). Controlling the structural and functional anisotropy of engineered cardiac tissues. Biofabrication.

[37] Pong T (2011). Hierarchical architecture influences calcium dynamics in engineered cardiac muscle. Exp. Biol. Med..

[38] Boudreau-Béland J (2015). Spatiotemporal stability of neonatal rat cardiomyocyte monolayers spontaneous activity is dependent on the culture substrate. PLoS One.

[39] Lanner JT, Georgiou DK, Joshi AD, Hamilton SL (2010). Ryanodine receptors: structure, expression, molecular details, and function in calcium release. Cold Spring Harb. Perspect. Biol..

[40] Feridooni HA, Dibb KM, Howlett SE (2015). How cardiomyocyte excitation, calcium release and contraction become altered with age. J. Mol. Cell. Cardiol..

[41] Daniels MCG, Naya T, Rundell VLM, de Tombe PP (2007). Development of contractile dysfunction in rat heart failure: hierarchy of cellular events. AJP Regul. Integr. Comp. Physiol..

