# Supplementary material for: Effect of patterned polyacrylamide hydrogel on morphology and orientation of cultured NRVMs
Source: Sci Rep. 2018 Aug 10;8:11991. doi: 10.1038/s41598-018-30360-6 (PMC6086831; doi:10.1038/s41598-018-30360-6)
Supplement: Supplementary file 1 — Supplementary Information [file 41598_2018_30360_MOESM1_ESM.pdf]

Supplementary Information

Effect of patterned polyacrylamide hydrogel on morphology and orientation of cultured NRVMs

I. Sanzari[a], E. J. Humphrey[b], F. Dinelli[c], C. Terracciano[b], T. Prodromakis[a]

[a]University of Southampton, Nanoelectronics & Nanotechnology Research Group, Electronics and Computer Science, Southampton, SO17 1BJ, UK

[b]National Heart and Lung Institute, Imperial College London, London, UK

[c]Consiglio Nazionale delle Ricerche (CNR), INO UOS 'A. Gozzini' Area della Ricerca di Pisa - S. Cataldo, via Moruzzi 1, I-56124 Pisa, Italy

E-mail: ilaria.sanzari@soton.ac.uk, e.humphrey13@imperial.ac.uk, franco.dinelli@ino.it, c.terracciano@imperial.ac.uk, T.Prodromakis@soton.ac.uk

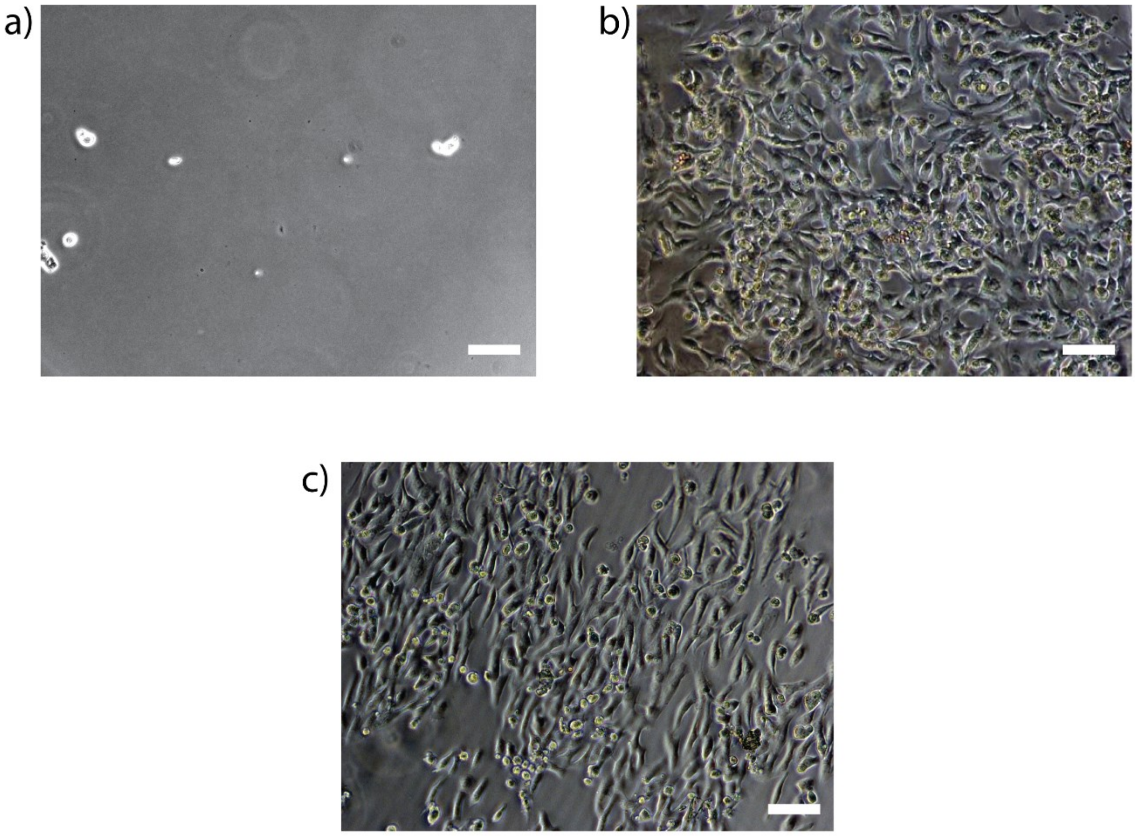

Figure S1. **Bright field images (4x magnification) of cell cultures on polyacrylamide (PAm) hydrogel samples with and without collagen patterns.** In (a) a pristine sample does not promote cell adhesion, demonstrating that PAm is not biocompatible. In (b) cells adhere with a random distribution on a PAm sample uniformly treated with collagen. In (c) cells align along the direction of collagen patterns. (White scale bar: 50 µm).
